# Towards enhanced telephone triage for chest pain: a Delphi study to define life-threatening conditions that must be identified

**DOI:** 10.1186/s12873-021-00553-w

**Published:** 2021-12-15

**Authors:** Ahmed Alotaibi, Richard Body, Simon Carley, Elspeth Pennington

**Affiliations:** 1 Division of Cardiovascular Sciences, Core Technology Facility, University of Manchester, 46 Grafton St, Manchester, M13 9WU UK; 2 College of Applied Medical Sciences, King Saud bin Abdulaziz University for Health Sciences, Riyadh, Saudi Arabia; 3 Emergency Department, Manchester University NHS Foundation Trust, Manchester, UK; 4 Faculty of Health, Social Care & Psychology, Manchester Metropolitan University, Manchester, UK; 5 North West Ambulance Service NHS Trust, Bolton, UK

**Keywords:** Prehospital, Triage, Telephone-triage, EMS, Chest pain, Life-threatening condition, Dispatch

## Abstract

**Background:**

Improving telephone triage for patients with chest pain has been identified as a national research priority. However, there is a lack of strong evidence to define the life-threatening conditions (LTCs) that telephone triage ought to identify. Therefore, we aimed to build consensus for the LTCs associated with chest pain that ought to be identified during telephone triage for emergency calls.

**Methods:**

We conducted a Delphi study in three rounds. Twenty experts in pre-hospital care and emergency medicine experience from the UK were invited to participate. In round I, experts were asked to list all LTCs that would require priority 1, 2, and 4 ambulance responses. Round II was a ranking evaluation, and round III was a consensus round. Consensus level was predefined at > = 70%.

**Results:**

A total of 15 participants responded to round one and 10 to rounds two and three. Of 185 conditions initially identified by the experts, 26 reached consensus in the final round. Ten conditions met consensus for requiring priority 1 response: oesophageal perforation/rupture; ST elevation myocardial infarction; non-ST elevation myocardial infarction with clinical compromise (defined, also by consensus, as oxygen saturation < 90%, heart rate < 40/min or systolic blood pressure < 90 mmHg); acute heart failure; cardiac tamponade; life-threatening asthma; cardiac arrest; tension pneumothorax and massive pulmonary embolism. An additional six conditions met consensus for priority 2 response, and three for priority 4 response.

**Conclusion:**

Using expert consensus, we have defined the LTCs that may present with chest pain, which ought to receive a high-priority ambulance response. This list of conditions can now form a composite primary outcome for future studies to derive and validate clinical prediction models that will optimise telephone triage for patients with a primary complaint of chest pain.

## Background

Chest pain is one of the most common reasons why patients call for emergency medical assistance (via 999, 911 or 112) [1, 2]. It is associated with various causes, which vary from life-threatening conditions to non-urgent conditions [3, 4]. Many different conditions could be considered to be life-threatening. These include acute coronary syndrome (ACS), aortic dissection, pulmonary embolism (PE) [3–5], tension pneumothorax and pericarditis [5]. On the contrary, other causes of chest pain do not require urgent attention, such as many musculoskeletal, respiratory, psychiatric and gastrointestinal aetiologies [6]. The majority of patients with a primary complaint of chest pain who are transported to hospital by emergency ambulance are ultimately diagnosed with non-cardiac disease and do not require hospitalisation for treatment of their condition [1, 7, 8]. Indeed, among patients who are admitted to hospital on suspicion of ACS, the actual prevalence of ACS is less than 20% [1, 7, 9].

Previous work has shown that there is currently only a low level of evidence to support the accuracy of medical telephone triage systems [10, 11] and the efficiency of current dispatch protocols [11, 12]. The definitions used in prehospital dispatch triage tools including Criteria Based Dispatch (CBD), Medical Priority Dispatch System (MPDS) [13, 14], and physician dispatch lack consensus [13]. There is also no consensus on the accepted level for over-triage or under-triage for medical emergency dispatch [10, 13].

Patients with chest pain are currently systematically over-triaged to avoid missing life-threatening conditions. Over-triage has been shown to occur in > 70% of cases in some systems [12, 13]. This increases ambulance resource consumption and contributes to Emergency Department crowding, which jeopardises patient safety and increases the cost of healthcare [1]. Despite this cautious approach, a research has demonstrated that only 46% of dispatch responses were safe, based on expert opinion [15].

The most significant limitation of research into dispatch accuracy might be the lack of consensus about precisely what conditions require an urgent response [13, 16]. One previous Delphi study was conducted to get consensus on cases that do not require an ambulance response. However, no results were reported [17]. Therefore, there is an urgent need to enhance telephone triage to more appropriately match the urgency of prehospital response to clinical need [18]. Prior to developing new telephone triage tools, it is imperative to define the life-threatening conditions (LTC) that telephone triage ought to identify, helping to ensure that patients with LTCs will receive an appropriately urgent prehospital response. Once we have defined the LTCs that require emergency response, we can run future studies to derive prediction models to accurately identify patients with those LTCs, using information that is available to telephone call handlers.

The aim of this research was to use expert consensus to define the LTCs associated with a primary complaint of non**-**traumatic chest pain, which would require priority 1, 2 and 4 ambulance responses.

## Methods

**Fig. 1:**
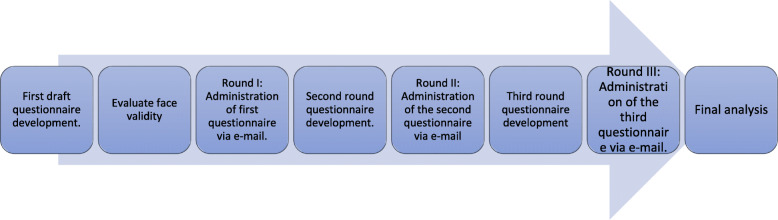
Flow diagram summarising the study protocol

In this consensus-based research, we applied a three-step Delphi technique. The process of this study consisted of generating ideas in the first round, ranking evaluation in the second round, and determining the presence or absence of consensus in the third round. Consensus threshold was predefined at > = 70% for either inclusion or exclusion. Figure 1 illustrates the overall study schedule.

The Delphi technique is a structured process for collecting and extracting information, where there is little or no evidence on a subject, from experts by distributing a series of questionnaires where the opinion feedback is controlled [19]. The technique is suggested for decision making agreement among anonymous experts [20]. Also, it has been used to define research priorities in Emergency Medical Services (EMS), but up to our knowledge, it hasn’t been conducted to define life-threatening emergencies associated with chest pain that require immediate ambulance response [21–25].

### Participant selection

Based on prior knowledge of potential experts in this field following work with the National Ambulance Research Steering Group (NARSG) and being part of multiple prehospital organizations, the study team agreed to nominate experts from various organizations and geographical areas within the UK. Those experts were identified using the following eligibility criteria: 1; Academic or clinician with expertise in emergency medicine or pre-hospital field 2; work experience of > = 3 years. The study team agreed that a minimum 3 years of experience would help to ensure that participants have sufficient clinical or academic expertise to demonstrate credibility and to make sound judgments about the conditions that require different priority ambulance responses.

As a result, twenty experts were identified by the study team and invited to participate in all rounds. Those identified experts were consultants in emergency medicine, academics or researchers in the prehospital field, paramedics, and emergency nurses. All experts invited had at least 3 years’ experience in the field. Variation of the opinions among the experts who were invited were expected based on their clinical expertise, research experience, career level and clinical role.

### Study protocol

In the first round, a web-based questionnaire was sent by e-mail to participants. The participants were asked to identify the conditions (or diagnoses) associated with chest pain that they believed would require priority 1 (7-min), 2 (18-min), and 4 (180-min) ambulance responses based on their experiences [18]. Priority 1 is described as life-threatening and defined as a time critical condition which requires immediate intervention or resuscitation; priority 2 is an emergency condition defined as potentially serious condition that might need urgent assessment, intervention or transport; priority 4 is less urgent which means the condition requires assessment with a possible need to transport the patient [18]. Additionally, the experts were asked to provide some demographic data including their speciality, years of experience, and region of employment. Reminders were sent to those who did not respond initially. A list of the conditions identified by the participants was prepared for inclusion in round II. Conditions that describe the same medical condition but with different terminology were grouped. Where the experts had suggested symptoms or patterns of clinical presentation (e.g. ‘chest pain with a history of heart disease’), we excluded the suggestion as these are potential predictors of outcome, rather than potential outcome variables for future research.

The second round of this study consisted of sending a second web-based questionnaire using Google Forms to all identified participants. The questionnaire included a list of identified conditions from round I. In this round, the participants were asked to rank each condition using a 5-point Likert Scale as Table 1 shows. The study team predetermined the inclusion and exclusion criteria for the final evaluation in the third round as described in Table 2. The conditions that meet inclusion and no consensus criteria was included in the list for round III. Any condition that met exclusion criteria in round II was removed and was considered not eligible for round III.

**Table 1:** Likert scale

1	2	3	4	5
Strongly disagree	Disagree	Neutral	Agree	strongly agree

**Table 2:** Consensus threshold

Round 2 & 3 Consensus criteria	
**Inclusion**	> 70% provide a positive result 4 or 5 on the scale.
**Exclusion**	> 70% provide a negative result 1 or 2 on the scale.
**No consensus**	Provided result for the condition doesn’t meet the inclusion or exclusion thresholds.

The third round of this study consisted of sending a third web-based questionnaire using Google Forms to all identified participants. In this final round, the questionnaire included conditions that already reached consensus to confirm the participants’ previous opinions or give them the opportunity to change it, which will increase the validity of those opinions. Also, it included conditions that had not achieved consensus to be further evaluated either for inclusion or exclusion. All conditions in this round were presented with the consensus percentages analysed from round II to show the experts which condition met inclusion criteria and non-consensus. Percentages showed how many experts chose (1, 2, 3, 4, or 5) from a 5-point Likert Scale. The participants responded to the questionnaire using a 5point Likert scale. Any condition that did not reach consensus for either inclusion or exclusion was then excluded. The final result showed all conditions that reached the threshold for inclusion or exclusion, and the proportion that did not reach consensus.

### Statistical analysis

We collated all responses from round I. The project steering team (consisting of the co-authors of this manuscript) merged responses that clearly referred to the same condition and removed responses that were clearly symptoms (e.g. sweating) rather than conditions. In rounds II and III, Likert scale responses were summarised using frequencies and percentages for each condition. Inclusion consensus threshold was defined as > = 70% of experts agreeing or strongly agreeing (4, or 5) on conditions using 5 points Likert Scale in rounds II, and III while exclusion consensus was defined as > = 70% of experts disagreeing or strongly disagreeing (2, or 1) Table 2. Data were analysed using Microsoft Excel 365.

## Results

### Demographics

In round I, a total of 20 participants were invited through email, of which 15 (75%) completed the online survey. The majority of the participants in this round were emergency doctors *n* = 6 (40%), and paramedics *n* = 5 (33.3%). The majority of participants had 16–20 years of experience (n = 6, 40%) (Table 3).

**Table 3:** summary of the expert’s demographics

Variable	Results n (%)		
	Round I	Round II	Round III
Clinical Role			
Emergency Doctor	6 (40%)	5 (50%)	3 (30%)
Emergency Nurse	2 (13.3%)	1 (10%)	2 (20%)
Paramedic	5 (33.3%)	4 (40%)	4 (40%)
Prehospital Academic or Researcher	1 (6.7%)	0	1 (10%)
Emergency Doctor and Clinical Academic	1 (6.7%)	0	
Years of Experience			
< 3 Years	0	0	0
3–5 Years	0	0	1 (10%)
6–10 Years	3 (20%)	3 (30%)	2 (20%)
11–15 Years	2 (13.3%)	1 (10%)	1 (10%)
16–20 Years	6 (40%)	3 (30%)	2 (20%)
21–25 Years	2 (13.3%)	2 (20%)	3 (30%)
26–30 Years	2 (13.3%)	1 (10%)	0
> 30 Years	0	0	1 (10%)
Region of current practice			
Scotland	1 (6.7%)	1 (10%)	0
Northern Ireland	0	0	0
Wales	0	0	0
North East	1 (6.7%)	0	0
North West	1 (6.7%)	1 (10%)	2 (20%)
Yorkshire and the Humber	0	0	0
West Midlands	3 (20%)	3 (30%)	2 (20%)
East Midlands	0	0	0
South West	3 (20%)	1 (10%)	1 (10%)
South East	1 (6.7%)	1 (10%)	2 (20%)
East of England	0	0	0
Greater London	5 (33.3%)	3 (30%)	3 (30%)

In round II, a total of 20 participants were invited by email. A total of 10 (50%) participants completed the online survey. Most of the participants in this round were emergency doctors n = 5 (50%), and paramedics *n* = 4 (40%). As in round I, the participants had a wide range of experience and the most common responses regarding years of experience were 16–20, and 6–10 years (*n* = 3, 30%) (Table 3).

In the final round, a total of 20 participants were invited by email. Out of the 20 invited participants, a total of 10 (50%) completed the online survey. Most of the participants in this round were paramedics n = 4 (40%) and emergency doctors n = 3 (30%). As in rounds I and II, the participants had a wide range of experience. The most common responses were 21–25 years (n = 3, 30%), 16–20, and 6–10 years (*n* = 2, 20%) (Table 3).

### Building consensus

**Fig. 2:**
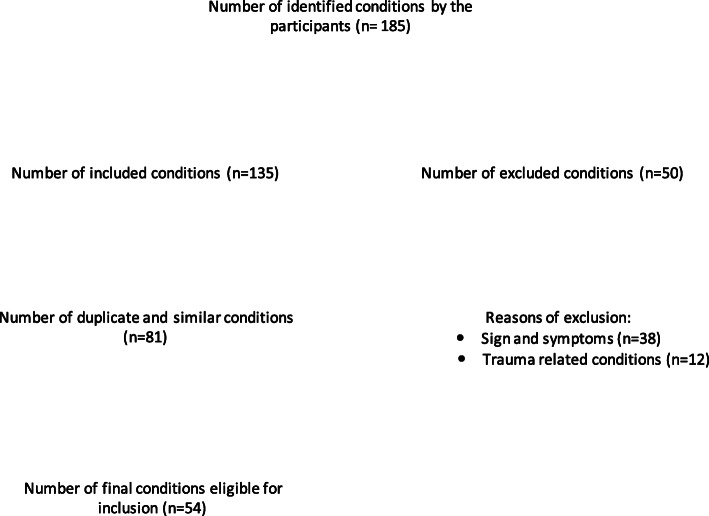
A flow chart of entire process for round I analysis

In round I, participants were asked to list all chest pain conditions that they felt would require priority 1, 2, and 4 ambulance responses. Fiftheen participants provided a total of 185 responses across all priorities surveyed. There were 73 conditions entered for priority 1, 70 for category 2, and 42 for category 4. After removing signs and symptoms or patterns of clinical presentation, there were 54 responses eligible for inclusion in round II. There were 18 chest pain conditions listed for priority 1, 27 conditions for priority 2, and 9 conditions for priority 4. A flow chart of the entire process for round I is shown in Fig. 2.

In round II, the participants were asked to rank the 54 chest pain conditions on a 5-point Likert scale. This resulted in 19 conditions meeting the pre-defined consensus threshold for inclusion (> = 70% of the responses were positive]4 or 5 on the Likert scale [), 2 meeting the pre-defined consensus threshold for exclusion (> = 70% of the response were negative]1 or 2 on the Likert scale [), and 33 conditions meeting the non-consensus threshold. Chest pain conditions that reached the inclusion and non-consensus threshold were included for progression to round III. As a result, 52 chest pain conditions were included in the final round after removing the two conditions that met exclusion consensus (Table 4).

**Table 4:** Round II analysis of conditions consensus

**Chest Pain conditions reaching inclusion consensus for priority 1**	**Inclusion %**
1. STEMI	90
Non-STEMI with clinical compromise	70
Acute left ventricular failure / acute heart failure	80
Aortic dissection	90
Cardiac tamponade	80
Life-threatening asthma	90
Cardiac arrest	100
Tension pneumothorax	90
Massive pulmonary embolism (pulmonary embolism with shock)	100
**Chest Pain conditions that did not achieve consensus for Priority 1**	
Submassive pulmonary embolism (pulmonary embolism without shock but with right heart strain)	
Arrhythmias	
Acute Coronary Syndrome (ACS)	
Acute left ventricular failure / acute heart failure	
Lower respiratory tract infection/chest sepsis	
Myocarditis	
Oesophageal perforation/rupture	
Pneumothorax (any)	
Pulmonary embolism (any, including subsegmental pulmonary embolism)	
**Chest Pain conditions reaching inclusion consensus for priority 2**	**Inclusion %**
STEMI	80
NSTEMI requiring immediate PCI	90
NSTEMI without clinical compromise	70
Acute left ventricular failure / acute heart failure	70
Cardiogenic shock	90
Abdominal aortic aneurysm	80
Lower respiratory tract infection with respiratory compromise	80
Pneumothorax with hypoxia	90
Ventricular tachycardia (with pulse)	90
Thoracic aortic aneurysm	70
**Chest Pain conditions reaching exclusion consensus for priority 2**	**Exclusion %**
Panic Attack	70
Gastro-oesophageal reflux disease (GORD)	100
**Chest Pain conditions that did not achieve consensus for priority 2**	
Acute coronary syndrome (ACS)	
Unstable angina	
Stable angina	
Arrhythmias (not peri-arrest)	
Biliary/peptic ulcer disease	
Asthma (not life threatening)	
COPD	
Lower respiratory tract infection/chest sepsis	
Pancreatitis	
Pulmonary embolism (any, including subsegmental)	
Pulmonary embolism with no clinical compromise	
Pericarditis	
Pleural effusion	
Pneumothorax (any)	
Supraventricular tachycardia (junctional tachycardia)	
**Chest Pain conditions that did not achieve consensus for priority 4**	
Chest infection	
Anxiety	
Costochondritis	
Gastro-oesophageal reflux disease (GORD)	
Lower respiratory tract infection (LRTI), sub-acute	
Pneumonia	
Musculoskeletal chest pain	
Pleurisy	
Shingles	

**STEMI:** ST-elevation myocardial infraction. **NSTEMI:** non-ST segment elevation myocardial infraction. **PCI:** percutaneous coronary intervention. **COPD:** chronic obstructive pulmonary disease

In the final round, the participants were asked to rank the 52 chest pain conditions on a 5-point Likert scale. This resulted in 26 chest pain conditions meeting the inclusion threshold (> = 70% of the response were positive]4 or 5 on the Likert scale [), zero chest pain conditions meeting the exclusion threshold (> = 70% of the response were negative]1 or 2 on the Likert scale [), and 26 meeting the non-consensus threshold. As planned a priori, chest pain conditions that reached the exclusion and non-consensus thresholds were removed. As a result, 26 chest pain conditions among all response categories were included (Table 5). After grouping the conditions, nineteen chest pain conditions were included. Among those nineteen, sixteen were considered to require category 1 or 2 ambulance responses (Table 6).

**Table 5:** Round III analysis of conditions consensus

**Chest Pain conditions reaching inclusion consensus for priority 1**	**Inclusion %**
STEMI	90
Non-STEMI with clinical compromise	90
Acute left ventricular failure / acute heart failure	90
Aortic dissection	90
Cardiac tamponade	100
Life-threatening asthma	100
Cardiac arrest	100
Tension pneumothorax	100
Massive pulmonary embolism (pulmonary embolism with shock)	100
Oesophageal perforation/rupture	80
**Chest Pain conditions that did not achieve consensus for priority 1**	
Submassive pulmonary embolism (pulmonary embolism without shock but with right heart strain)	
Arrhythmias	
Acute Coronary Syndrome (ACS)	
Acute left ventricular failure / acute heart failure	
Lower respiratory tract infection/chest sepsis	
Myocarditis	
Pneumothorax (any)	
Pulmonary embolism (any, including subsegmental pulmonary embolism)	
**Chest Pain conditions reaching inclusion consensus for priority 2**	**Inclusion %**
STEMI	90
NSTEMI requiring immediate PCI	90
NSTEMI without clinical compromise	90
Acute left ventricular failure / acute heart failure	80
Cardiogenic shock	90
Abdominal aortic aneurysm	90
Lower respiratory tract infection with respiratory compromise	90
Pneumothorax with hypoxia	90
Ventricular tachycardia (with pulse)	90
Thoracic aortic aneurysm	90
Acute coronary syndrome (ACS)	100
Unstable angina	70
Supraventricular tachycardia (junctional tachycardia)	80
**Chest Pain conditions that did not achieve consensus for priority II**	
Stable angina	
Arrhythmias (not peri-arrest)	
Biliary/peptic ulcer disease	
Asthma (not life threatening)	
COPD	
Lower respiratory tract infection/chest sepsis	
Pancreatitis	
Pulmonary embolism (any, including subsegmental)	
Pulmonary embolism with no clinical compromise	
Pericarditis	
Pleural effusion	
Pneumothorax (any)	
**Chest Pain conditions reaching inclusion consensus for priority 4**	**Inclusion %**
Chest infection	70
Gastro-oesophageal reflex disease (GORD)	70
Lower respiratory tract infection (LRTI), sub-acute	80
**Chest Pain conditions that did not achieve consensus for priority 4**	
Anxiety	
Costochondritis	
Pneumonia	
Musculoskeletal chest pain	
Pleurisy	
Shingles	

**STEMI:** ST-elevation myocardial infraction. **NSTEMI:** non-ST segment elevation myocardial infraction. **PCI:** percutaneous coronary intervention. **COPD:** chronic obstructive pulmonary disease

**Table 6:** final inclusion result per priority after modification

**Priority 1**	**Inclusion %**
Oesophageal perforation/rupture	80%
STEMI	90%
NSTEMI with clinical compromise	90
acute left ventricular failure/ acute heart failure	70
Aortic Dissection	90
Cardiac tamponade	100
Life-threatening Asthma	100
Cardiac Arrest	100
Tension Pneumothorax	100
Massive pulmonary embolism (pulmonary embolism with shock)	100
**Priority 2**	
Acute coronary syndrome (ACS)	100
Supraventricular tachycardia (junctional tachycardia)	80
Abdominal Aortic Aneurysm	90
Lower respiratory tract infection with respiratory compromise	90
Pneumothorax with hypoxia	90
Ventricular tachycardia (with pulse)	90
**Priority 4**	
Chest infection	70
Gastro-oesophageal reflux disease (GORD)	70
Lower respiratory tract infection (LRTI), sub-acute	80

**STEMI:** ST-elevation myocardial infraction. **NSTEMI:** non-ST segment elevation myocardial infraction

**Fig. 3:**
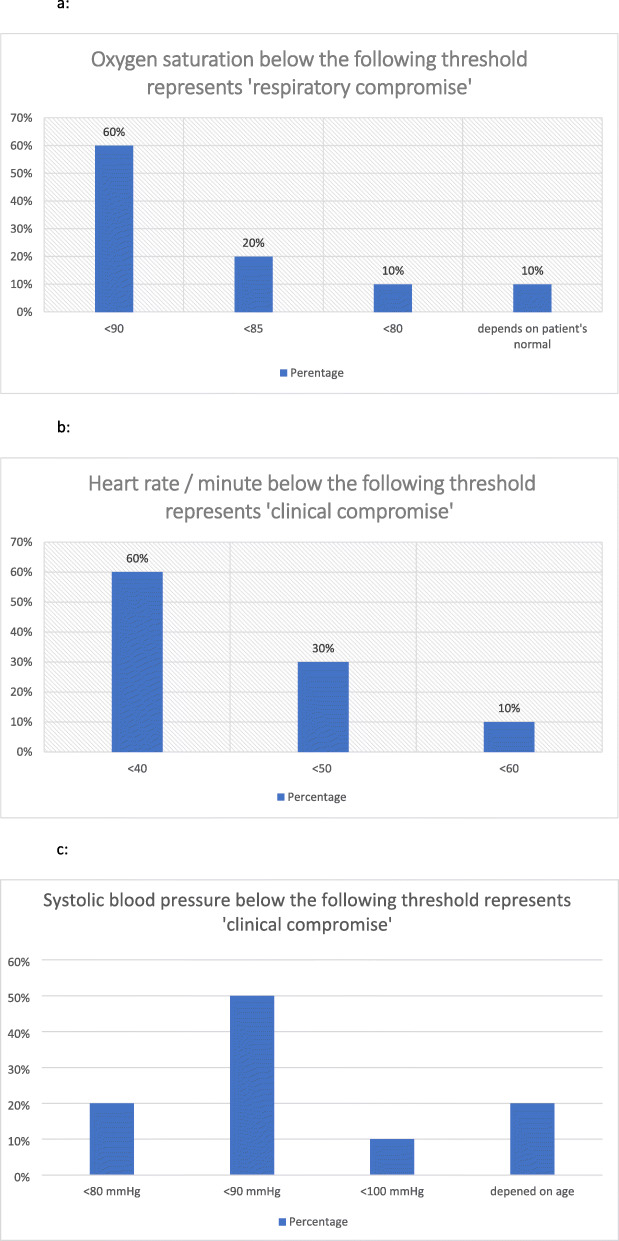
**a** Clinical compromise related to Oxygen saturation. **3b** Clinical compromise related to heart rate. **3c** Clinical compromise related to blood pressure

In round I, the following two conditions were listed by participants: ‘NSTEMI with clinical compromise’, and ‘NSTEMI without clinical compromise’. ‘Clinical compromise’ was not defined by participating experts. Therefore, the study team agreed to add three questions to define ‘clinical compromise’ for adult paitents in round III. Those were multiple choice questions regarding thresholds for defining ‘clinical compromise’ using blood pressure, heart rate and oxygen saturation. All ten participants answered the questions. As a result, the definition of clinical compromise met consensus criteria at the following thresholds: oxygen saturation < 90% (Fig. 3a), heart rate < 40 beats per minute (Fig. 3b), and systolic blood pressure < 90 mmHg (Fig. 3c).

## Discussion

In this work, we have used expert consensus to define the LTCs that require ambulance responses with different priorities, sixteen of which were deemed to require a category 1 or 2 ambulance response. Given the evidence of systematic over- and under-triage with current telephone triage systems, there is a clear need to derive improved models for telephone triage of patients with chest pain. Our findings should inform that work, as future telephone triage models should be able to correctly identify patients with the LTCs defined by our expert panel. This can be achieved by using the list of LTCs that we have defined as a composite primary outcome for future studies to derive and validate prediction models that will optimise telephone triage for this patient group.

By informing the design of such future research, our work will help to ensure that priority 1 ambulance responses are reserved for patients who need it most, optimising efficiency and ensuring cost-effectiveness. We have also identified specific conditions that may require a less urgent (priority 4) ambulance response. This is a vital first step towards developing new models to enhance telephone triage for patients with chest pain, because we have identified which conditions (or diagnoses) such a prediction model should be able to predict, using information that is available to call handlers.

There were some notable findings in our research. For example, both STEMI and NSTEMI with clinical compromise achieved consensus for requiring a priority 1 (8-min) ambulance response. Under current EMS dispatch systems in the UK, patients suspected to have either of these conditions would receive a category 2 ambulance response. Thus, our expert panel assigned a higher priority to those conditions than is currently used in clinical practice. This could potentially lead to more patients requiring an immediate priority 1 ambulance response, which could cause concern about increasing resource utilisation. However, by deriving and validating a prediction model to accurately identify the LTCs identified by our expert panel, we would hope that the specificity of triage will be increased overall, reducing the number of patients who receive high-priority ambulance responses despite not having an LTC, and therefore optimising both safety and efficiency.

This study has some limitations; the response rates in rounds II and III were relatively low (50%) in comparison to round I (75%). The reason might be the impact of Covid-19 as the participants are healthcare workers and were busy during the pandemic. However, our final sample size is generally considered acceptable for a study of this nature [20]. Further, participants in this study were all from the UK so our results may not be applicable for other countries.

## Conclusion

Using expert consensus, we have defined the chest pain conditions that require different ambulance priority responses. The final result includes 16 chest pain conditions which can be used as a national definition for LTC associated with chest pain, These results could be used as a composite primary outcome in future research to derive and validate clinical prediction models to optimise telephone triage for patients with a primary complaint of chest pain.

## Data Availability

All data used for this study are reported within the article.

## Abbreviations

ACS: Acute coronary syndrome
PE: Pulmonary embolism
CBD: Criteria based dispatch
MPDS: Medical priority dispatch system
LTC: Life-threatening condition
EMS: Emergency medical services
STEMI: ST-elevation myocardial infraction
NSTEMI: Non-ST segment elevation myocardial infraction
PCI: Percutaneous coronary intervention
COPD: Chronic obstructive pulmonary disease
